# The influence of inter-particle forces on diffusion at the nanoscale

**DOI:** 10.1038/s41598-019-48754-5

**Published:** 2019-09-03

**Authors:** Francesco Giorgi, Diego Coglitore, Judith M. Curran, Douglas Gilliland, Peter Macko, Maurice Whelan, Andrew Worth, Eann A. Patterson

**Affiliations:** 1 Faculty of Science and Engineering, University of Liverpool, Liverpool, L69 3GH United Kingdom; 2 CNR Nanotec, Lecce, 73100 Italy; 3 European Commission, Joint Research Centre (JRC), Ispra, 21027 Italy

**Keywords:** Nanoparticles, Microscopy

## Abstract

Van der Waals and electrostatic interactions are the dominant forces acting at the nanoscale and they have been reported to directly influence a range of phenomena including surface adhesion, friction, and colloid stability but their contribution on nanoparticle diffusion dynamics is still not clear. In this study we evaluated experimentally the changes in the diffusion coefficient of nanoparticles as a result of varying the magnitude of Van der Waals and electrostatic forces. We controlled the magnitude of these forces by varying the ionic strength of a salt solution, which has been shown to be a parameter that directly controls the forces, and found by tracking single nanoparticles dispersed in solutions with different salt molarity that the diffusion of nanoparticles increases with the magnitude of the electrostatic forces and Van der Waals forces. Our results demonstrate that these two concurrently dynamic forces play a pivotal role in driving the diffusion process and must be taken into account when considering nanoparticle behaviour.

## Introduction

**Figure 1:**
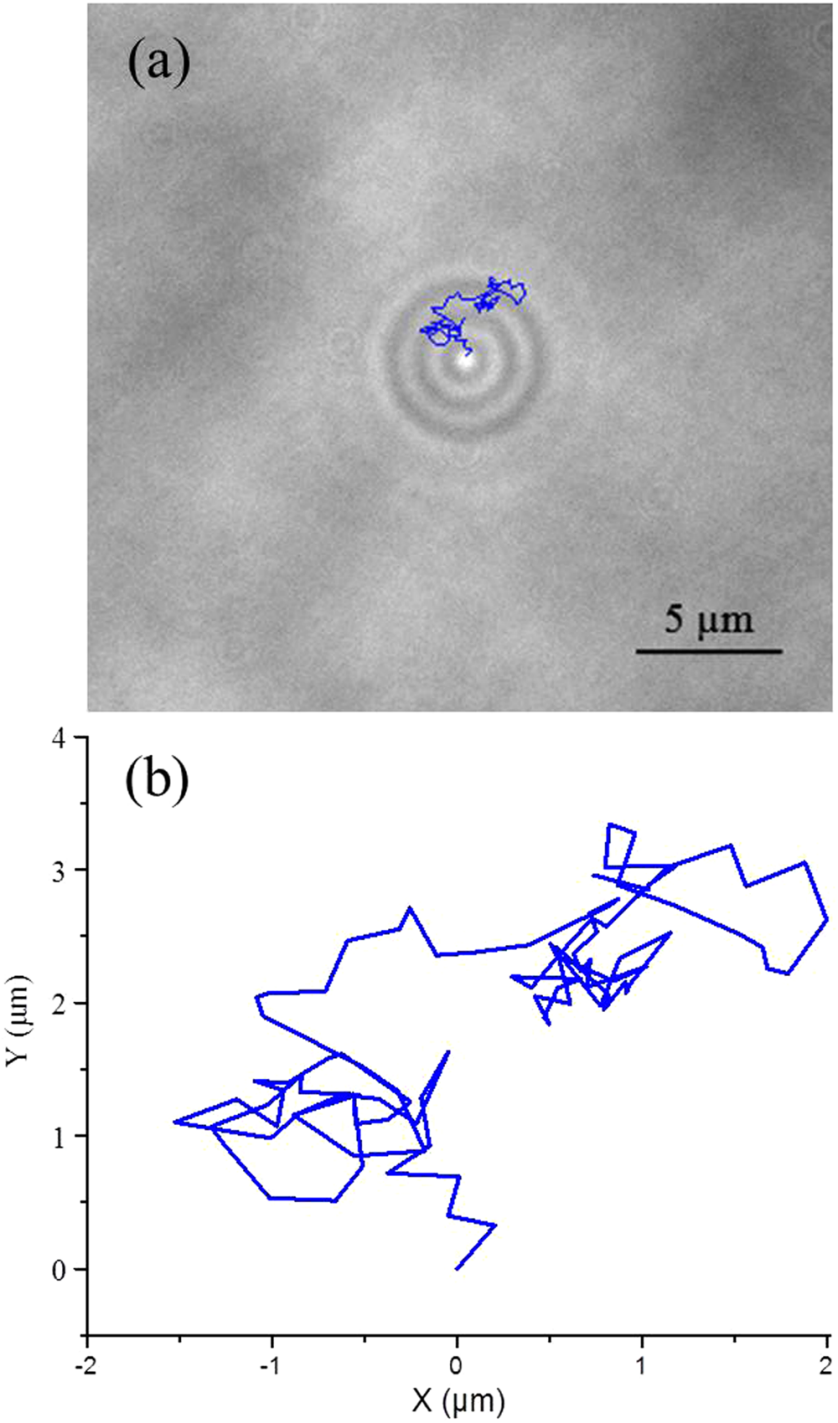
(**a**) Photograph of a caustic generated by a 50 nm gold nanoparticle in water taken with the optical microscope used in this work adjusted for Kohler illumination and closing the condenser field aperture to its minimum following method of Patterson and Whelan^9^ with its 2d random walk over a period of 3 seconds superimposed and (**b**) a plot of the same walk.

Gold nanoparticles have attracted a growing interest in the scientific community in recent years, especially for biological and medical applications. However, there is a lack of knowledge about the mechanisms that regulate their diffusion through liquid media. It is well-known that two of the main forces acting on charged nanoparticles dispersed in a medium are Van der Waals and electrostatic forces, but their actual contribution to particle diffusion has not been experimentally investigated. The local ionic concentration, a parameter that directly influences the magnitude of electrostatic repulsion forces between particles, has been reported to strongly influence the intrinsic properties of dispersed colloids. A number of studies have shown that the addition of salt negatively affects the colloidal stability of nanoparticles in solution, leading to aggregation and settling^1,2^ because of the formation of a double-layer on the surface of each particle. The first layer consists of ions absorbed onto the surface of a particle while the second one is composed of counter ions dissolved in solution which start to surround the particles attracted by its surface charge. This second layer shields the original charge of the particle and reduces the electrostatic repulsion forces acting between particles^3^. Other phenomena, like the apparent size reduction, has been reported in the literature as a result of nanoparticles’ exposure to salt solution^4^. Nevertheless, little is known about the contribution of the ionic strength of the solution to the diffusion coefficient of nanoparticles before the salt reaches a critical concentration leading to fast aggregation rate. The majority of the studies in the literature evaluate nanoparticle diffusion taking into account only the physical parameters identified by the Stoke-Einstein equation^5^ including the viscosity^6^ and temperature^7^ of the medium as well as the nanoparticles’ size^8^, ignoring the potential effects of electrochemical forces. In this study, we investigate whether and how the ionic strength of the solution influences the diffusion of gold nanoparticles by tracking single particles using an optical microscope to visualize the caustic generated by individual nanoparticles, exploiting the technique proposed by Patterson and Whelan (Fig. 1)^9^. The results obtained suggest that for salt molarity below 50 mM NaCl, the diffusion coefficient of the particles tested consistently increased with increasing molarity, confirming that electrostatic forces, Van der Waals forces and the nanoparticle-ion interactions should be accounted for when considering the diffusion dynamics of nanoparticles.

## Results and Discussion

**Figure 2:**
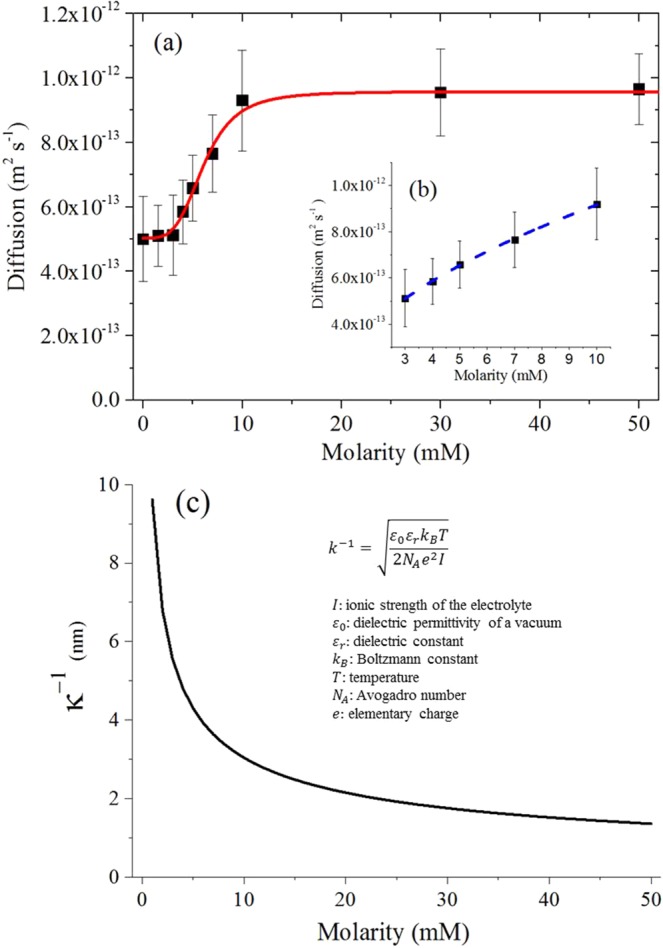
(**a**) Experimental diffusion coefficients for 50 nm diameter gold nanoparticles as a function of NaCl solution molarity fitted by Hill function (red continuous line). (**b**) Detail of the diffusion coefficient for 50 nm gold nanoparticle dispersed in solutions ranging from 3 mM to 10 mM of NaCl fitted by a square root function (blue dotted line). (**c**) Debye length trend as a function of NaCl solution molarity calculated from its theoretical equation^15^.

Ultraviolet and visible (UV-Vis) absorption spectroscopy (U-2900, Hitachi) was used to evaluate the colloidal stability of the nanoparticle solution tested within our experimental timescale. The results obtained confirmed the absence of aggregates and clusters for NaCl molarity up to 50 mM, hence this value was chosen as maximum salt concentration for our single nanoparticle tracking (Fig. S1). This was expected considering results reported by others in the literature for the same nanoparticles and salt species^1^ and also by the fact that the method used here to track single particles using an optical microscope can easily distinguish between single particles and clusters^9^. The values for the diffusion coefficient of 50 nm gold nanoparticles dispersed in NaCl solutions ranging from 0 to 50 mM are shown in Fig. 2a. It can be observed that the overall trend of the diffusion coefficient can be effectively described by fitting the experimental data obtained with the Hill function, which has been widely used to describe experimental phenomenon when the relationship between two sets of variables seems saturable and nonlinear^10^. The empirical relationship describing the increasing trend in diffusion as a function of the NaCl molarity can be directly derived by adapting the Hill equation:

1 $$D(M)={D}_{water}+\frac{({D}_{max}-{D}_{water}){M}^{n}}{{k}^{n}+{M}^{n}}$$

where *D*_*water*_ and *D*_*max*_ are the minimum (in water) and maximum values respectively of the diffusion coefficient for nanoparticles obtained from experiment, *n* is the Hill coefficient, *k* is the molarity value at which the diffusion coefficient reaches the midpoint between its maximum and minimum and *M* is the molarity of the solution. The Hill coefficient, *n*, is used in the literature as an interaction coefficient reflecting the cooperativity in a binding process between two or more species of molecule. A value greater than 1 qualitatively indicates positive cooperative binding^11^. For example, considering a system where one receptor molecule of species A can bind to two ligand molecules of species B, the binding process can be considered cooperative if the binding of the first molecule of B to A increases the binding affinity of the second B molecule. The value of the Hill coefficient found experimentally in this study is equal to 3.70 and can be used in our scenario to describe the increased electrical affinity between the nanoparticle surface (species A) and the counter ions (species B) in solution, i.e. the number of counter ions electrically bound to nanoparticle surface because of electrostatic interaction increases as a function of the molarity of the solution until a maximum value is reached at a salt concentration of 10 mM at which the counter ions completely cover the nanoparticles surface.

In our test scenario, there are no significant differences in the value of diffusion coefficient up to a salt concentration of 3 mM, suggesting that the ionic strength of the solution and the number of ions electrically bound to nanoparticle surface are not enough to influence the particle dynamics. The diffusion values exhibited by these solutions (approximately 5 × 10^−13^ m^2^ s^−1^) are in good agreement with the values found by Coglitore *et al*. for the diffusion of gold nanoparticles in deionised water^12^. At higher levels of NaCl concentration there is an increase in the particle diffusion, which tends to the saturation value of 10^−12^ m^2^ s^−1^. The increasing trend observed contradicts the theoretical prediction of the Stoke–Einstein equation. In fact, increasing the NaCl concentration in the solution leads to a small but constant increase in the viscosity of the solution. At a NaCl concentration of 50 mM, the solution exhibits a dynamic viscosity which is approximately 0.5% greater than the viscosity of pure de-ionised water^13^. This viscosity increase should cause a small but proportional decrease in the diffusion coefficient of the particles, or at least should not support any increase in the diffusion, because the diffusion coefficient is inversely proportional to solution viscosity, according to the Stoke-Einstein equation^5^.

This increasing behaviour can be explained by considering the effects of the ions on the nanoparticles and solution properties. When the negatively charged nanoparticles are dispersed in a salt solution, Na^+^ ions start to surround their spherical surface, and the number of ions electrically bound to the surface will increase as a function of the solution molarity. Na^+^ ions exhibit a value for the diffusion coefficient in water at 303 K of 1.4 × 10^−11^ m^2^ s^−1 ^^14^, which is two orders of magnitude larger than for the gold nanoparticles in deionised water, so it is reasonable to assume that when a critical density of ions electrically bound to the particle is reached, they will form a corona and contribute actively to the dynamics of the particles causing them to diffuse more quickly.

Moreover, the density of ions surrounding each particle controls directly the magnitude of the electrostatic interactions through variations in the Debye length, which is a measure of the electrical double layer thickness^15^. According to the well-known DLVO theory, the dominant forces acting at the nanoscale between particles are the electrostatic forces and the Van der Waals forces. These two forces are independent and opposite in action, i.e. electrostatic forces repel and Van der Waals forces attract particles, therefore they can be superimposed to obtain a good approximation of the net interaction force between two particles^16^. However, as the particle size and the distance between particles decreases, the non-additivity of the forces acting on the nanoparticles emerges. In other words, it becomes impossible to decompose the net interaction forces acting between particles into separate additive contributions in order to understand the influence of each force independently from the others^17^. Hence, in this study we decided to investigate the diffusion changes observed as a result of the action of Van der Walls and electrostatic forces together. At a low ionic strength, the Debye length is large because of the small concentration of counter ions surrounding the particle and the electrostatic repulsion forces are dominant relative to Van der Waals attraction forces. A higher ionic strength causes a more effective shielding of the original charge of the particle and a reduction of the Debye length, leading to a decrease of the electrostatic repulsion forces. Hence, the net interaction force between particles becomes slightly attractive, because of the constant contribution of the Van der Waals forces which are insensitive to the ionic strength. Moreover, the resultant net attractive interaction force causes the nanoparticles to be closer to each other, leading to an increased magnitude of the Van de Waals forces acting between particles, which are distance dependant. Our results demonstrate that these concurrent effects (reduction of electrostatic repulsion and increase of Van der Waals attraction) contribute actively to the nanoparticle diffusion process before aggregation occurs at higher salt molarity and that there is a direct correlation between the variation of the diffusion coefficient of the gold nanoparticle and the variation of the Debye length. Figure 2b shows the detail of the increase in the diffusion coefficient exhibited by the 50 nm gold nanoparticles dispersed in solutions with concentrations between 3 mM to 10 mM NaCl. It can be seen that the increased rate of the diffusion of the nanoparticle in this molarity range follows a square root trend, which is exactly the inverse of the trend in Debye length as illustrated in Fig. 2c. For concentrations between 0 and 10 mM NaCl, there is a significant reduction in the Debye length and hence electrostatic repulsion, leading to a noticeable increase in their diffusion through the medium. A further increase in the salt concentration (10 mM to 50 mM NaCl) causes an increasingly smaller reduction in the Debye length and the electrostatic repulsion forces, therefore the diffusion of the nanoparticles saturates and tends to a maximum value of 10^−12^ m^2^ s^−1^. The increasing trend of diffusion coefficient demonstrated in this study may also resemble the salt-induced transition from the slow to the fast aggregation regime for nanoparticles^18^. However, with the optical technique used in this work it was possible to easily distinguish between single nanoparticles and clusters^9^ so that no aggregation occurred during the experiments, which was confirmed by spectral analysis (Fig. S1). Moreover, for gold nanoparticles in NaCl solution, the transition from the slow to fast aggregation regime has been reported to appear at a salt concentration of 75 mM^1^, which is higher than the maximum molarity value (50 mM) used in this work.

**Figure 3:**
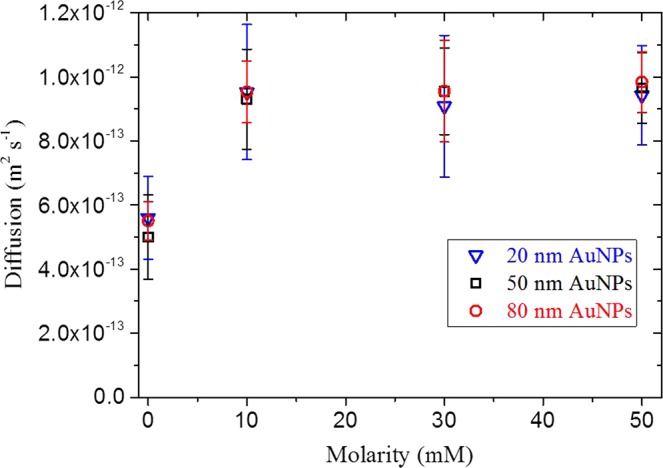
Experimental diffusion coefficients for 20 nm, 50 nm and 80 nm diameter gold nanoparticles as function of NaCl solution molarity.

The dynamics of 20 nm and 80 nm diameter gold nanoparticles dispersed in NaCl solutions was also evaluated and compared with the diffusion behavior exhibited by the 50 nm diameter gold nanoparticles in Fig. 3. Both the 20 nm, 50 nm and 80 nm exhibit comparable initial (between 0 and 3 mM of NaCl) and final (between 10 and 50 mM of NaCl) diffusion coefficient, demonstrating that under our test conditions the diffusion of the nanoparticles was found to be independent of particle size. Recent studies have demonstrated the independence of the diffusion coefficient on particle size for gold and polystyrene nanoparticles in a simple fluid for particles of diameter less than 150 nm at concentrations below 10^9^ particles ml^−1 ^^12^. In our study, this size-independence can be explained by the fact that Van der Waals and electrostatic forces both scale linearly with the particle radius, suggesting that an increase or decrease of particle size causes a comparable change to both these competing forces^19^.

## Conclusion

In summary, the data presented within this paper demonstrate that the diffusion of gold nanoparticles is strongly influenced by the magnitude of the electrostatic and Van der Waals forces, controlled by the variation of the ionic strength of the solution. The effect of these two forces was found to be dominant with respect to others factors, such as particle size, which was found to be negligible within the tested range from 20 nm to 80 nm and a concentration of 5 × 10^8^ particles ml^−1^. These findings have potential implications, for example, for understanding and predicting the behavior of nanoparticles dispersed in biological media which typically contain NaCl in solution^20^. The ionic strength of the biological medium can therefore affect nanoparticle diffusion which has been shown to influence cellular uptake and toxicity^21^. Hence, future investigations should take into account the complexity of the diffusion process and parameters like the ionic strength of the solution; in addition, the electrostatic and Van der Walls interactions should be considered in order to correctly evaluate the dynamics of the nanoparticles.

## Material and Methods

Spherical negatively charged gold nanoparticles were purchased from BBI Solutions, with a nominal diameter of 20 nm, 50 nm and 80 nm. The as-supplied concentrations were reduced by adding the concentrate to deionised water as appropriate to obtain a constant working concentration of 5 × 10^8^ particles ml^−1^. Nanoparticles were dispersed in solutions with ionic strength ranging from 0 to 50 mM, achieved by mixing deionised water and Sodium Chloride (Sigma Aldrich). A mixer (Vortex-Genie 2 G560E, Scientific Industries Inc.) was employed to obtain a uniform particle distribution. Four independent solutions were prepared for each NaCl molarity and in each salt solution at least six particles were tracked so that the diffusion data presented are average values with standard deviations. The experimental value for the diffusion of each particle was calculated from its Mean Square Displacement (MSD)^22^, and the single particle tracking analysis was performed following the method used by Coglitore *et al*.^12^ in a standard inverted optical microscope (Axio Observer.Z1 m, Carl Zeiss) mounted on antivibration feet (VIBe, Newport) to isolate the sample from the environment. The solution temperature was maintained at a constant T = 303 K during each test using a heated microscope stage (Heatable universal mounting frame KH S1, Pecon GmbH) connected to a controller (Tempcontroller 2000-2, Pecon GmbH) and monitored, for further confirmation of the temperature value, by two thermocouples.

## Supplementary information

Supporting Information - The influence of inter-particle forces on diffusion at the nanoscale

## References

[1] Pamies, R. *et al*. Aggregation behaviour of gold nanoparticles in saline aqueous media. *J*. *Nanopart*. *Res*. **16**(4) (2014).

[2] Liu J (2012). Influence of surface functionalization and particle size on the aggregation kinetics of engineered nanoparticles. Chemosphere..

[3] Pfeiffer, C. *et al*. Interaction of colloidal nanoparticles with their local environment: the (ionic) nanoenvironment around nanoparticles is different from bulk and determines the physico-chemical properties of the nanoparticles. *J*. *R*. *Soc*. *Interface*. **11** (2014). 10.1098/rsif.2013.0931 PMC4032524 24759541

[4] Rehbock C, Merk V, Gamrad L, Streubel R, Barcikowski S (2013). Size control of laser-fabricated surfactant-free gold nanoparticles with highly diluted electrolytes and their subsequent bioconjugation. Phys. Chem. Chem. Phys..

[5] Miller C (1921). The Stokes-Einstein law for diffusion in solution. Proc. Roy. Soc..

[6] Wong, K., Chen, C., Wei, K., Roy, V. A. L. & Chathoth, S. M. Diffusion of gold nanoparticles in toluene and water as seen by dynamic light scattering. *J*. *Nanopart*. *Res*. **17**(3) (2015).

[7] Qiao Z, Feng H, Zhou J (2014). Molecular dynamics simulations on the melting of gold nanoparticles. Phase Transitions..

[8] Li, Z. Critical particle size where the Stokes-Einstein relation breaks down. *Phys*. *Rev*. *E Stat*. *Nonlinear*. *Soft*. *Matter*. *Phys*. **80** (2009). 10.1103/PhysRevE.80.061204 20365158

[9] Patterson, E. A. & Whelan, M. P. Tracking nanoparticles in an optical microscope using caustics. *Nanotech*. **19**(10) (2009). 10.1088/0957-4484/19/10/105502 21817700

[10] Goutelle S (2008). The Hill equation: A review of its capabilities in pharmacological modelling. Fundam. Clin. Pharmacol..

[11] Santillán M (2008). On the use of the hill functions in mathematical models of gene regulatory networks. Math. Model Nat. Phenom..

[12] Coglitore, D., Edwardson, S. P., Macko, P., Patterson, E. A. & Whelan, M. Transition from fractional to classical Stokes-Einstein behaviour in simple fluids. *R*. *Soc*. *Open Sci*. **4**(12) (2017). 10.1098/rsos.170507 PMC5749985 29308217

[13] Zhang H, Han S (1996). Viscosity and density of water + sodium chloride + potassium chloride solutions at 298.15 K. J Chem Eng Data..

[14] Kim JS, Wu Z, Morrow AR, Yethiraj A, Yethiraj A (2012). Self-diffusion and viscosity in electrolyte solutions. J. Phys. Chem. B..

[15] Zhang W (2014). Nanoparticle aggregation: Principles and modelling. Adv. Exp. Med. Biol..

[16] He Z, Alexandridis P (2015). Nanoparticles in ionic liquids: Interactions and organization. Phys. Chem. Chem. Phys..

[17] Batista, C. A. S., Larson, R. G. & Kotov, N. A. Nonadditivity of nanoparticle interactions. *Science*, **350**(6257) (2015). 10.1126/science.1242477 26450215

[18] Oncsik T, Trefalt G, Borkovec M, Szilagyi I (2015). Specific ion effects on particle aggregation induced by monovalent salts within the Hofmeister series. Langmuir..

[19] Hotze EM, Phenrat T, Lowry GV (2010). Nanoparticle aggregation: Challenges to understanding transport and reactivity in the environment. J. Environ. Qual..

[20] Huang J (2011). Inhibitory effects and mechanisms of physiological conditions on the activity of enantiomeric forms of an α-helical antibacterial peptide against bacteria. Peptides..

[21] Cho EC, Zhang Q, Xia Y (2011). The effect of sedimentation and diffusion on cellular uptake of gold nanoparticles. Nat. Nanotechnol..

[22] Michalet X (2010). Mean square displacement analysis of single-particle trajectories with localization error: Brownian motion in an isotropic medium. Phys. Rev. E Stat. Nonlinear. Soft. Matter. Phys..

