# Supplementary material for: The influence of inter-particle forces on diffusion at the nanoscale
Source: Sci Rep. 2019 Sep 3;9:12689. doi: 10.1038/s41598-019-48754-5 (PMC6722115; doi:10.1038/s41598-019-48754-5)
Supplement: Supplementary file 1 — Supporting Information - The influence of inter-particle forces on diffusion at the nanoscale [file 41598_2019_48754_MOESM1_ESM.docx]

Supporting Information

The influence of inter-particle forces on diffusion at the nanoscale

*Francesco Giorgi^†^, Diego Coglitore^‡^, Judith M. Curran^†^, Douglas Gilliland^§^, Peter Macko^§^, Maurice Whelan^§^, Andrew Worth^§^, and Eann A. Patterson^†^.*

^†^ School of Engineering, University of Liverpool, Liverpool L69 3GH, United Kingdom

^‡^ CNR Nanotech, Lecce 73100, Italy

**^§^** European Commission, Joint Research Centre (JRC), Ispra 21027, Italy

Keywords: Diffusion; gold nanoparticles; ionic strength; Van der Waals forces; electrostatic forces; Debye length.

**List of supporting material:**

**S1.** Ultraviolet and visible absorption spectroscopy analysis

**S1. Ultraviolet and visible absorption spectroscopy analysis**

We evaluated the colloidal stability of gold nanoparticles solutions as a function of NaCl molarity by using UV-vis absorption spectroscopy (Hitachi, U-2900). Monodispersed 50 nm gold nanoparticles solutions exhibit a narrow absorption band with a maximum at 520 nm and the absorbance peak value is directly correlated to the concentration of monodispersed particles in solution^[1]^. Figure S1 shows the absorption spectra of the gold nanoparticles solution tested in our work. The wavelength at which the maximum absorption peak occurs is directly related to nanoparticle size. As the diameter of the nanoparticles increases, the absorption peak red-shift to longer wavelength. If aggregation of nanoparticles occurs, the spectrum modifies, exhibiting an increased UV-visible intensity at longer wavelengths and a broadening of the absorption peak, because of the formation of nanoparticle aggregates of different size^[2]^.


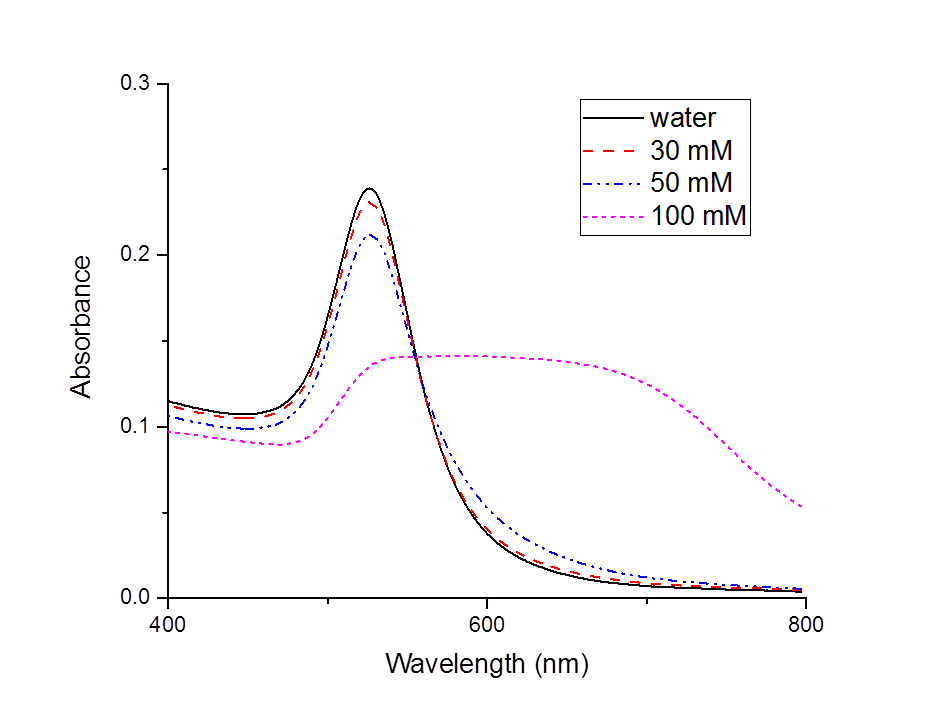


**Figure S1.** Absorption spectra of 50 nm gold nanoparticles (concentration 5 x 10^8^ particles ml^-1^) dispersed in NaCl solutions ranging from 0 to 100 mM.

As expected, the addition of NaCl in solutions of nanoparticles induces changes in the absorption spectra, but up to 50 mM of NaCl, the majority of the particles are still monodispersed. A further increase of the NaCl leads to the formation of nanoparticles aggregates of different size, resulting in the broadening of the spectrum and in the reduction of the absorption peak, meaning that the majority of nanoparticles in solution are not monodispersed. Hence, we performed our single particle tracking analysis within this molarity range. These results are confirmed by others in the literature with the same nanoparticles and salt species^[3]^. The method used in this work to track single particles using an optical microscope can easily distinguish between single particles and the occasional cluster that might be encountered in this low molarity range^[4]^.

**References**

[1] W. Haiss, N. T. K. Thanh, J. Aveyard, D. G. Fernig, Anal. Chem. **2007**, *79*, 4215.

[2] X. Huang, M. A. El-Sayed, J. Adv. Res. **2010**, *1*, 13.

[3] R. Pamies, J. G. H. Cifre, V.F. Espín, M. Collado-González, F. G. D. Baños, J. G. De La Torre, J. Nanopart. Res. **2014**, *16*.

[4] E. A. Patterson, M. P. Whelan, Small **2008**, *4*, 1703.
